# Metal complexes in medicine: structural scaffolds *vs.* functional centres

**DOI:** 10.1039/d6sc01182a

**Published:** 2026-03-24

**Authors:** Nicolás Montesdeoca, Johannes Karges

**Affiliations:** a Department of Biophysics, Faculty of Medicine, Ruhr University Bochum Universitätsstrasse 150 44801 Bochum Germany johannes.karges@ruhr-uni-bochum.de +49 2343224187 https://www.kargesgroup.ruhr-uni-bochum.de/; b Center for Protein Diagnostics (PRODI), Ruhr University Bochum Gesundheitscampus 4 44801 Bochum Germany

## Abstract

Metal complexes occupy a distinctive position in medicinal chemistry by combining well-defined three-dimensional structures with tuneable reactivity that cannot be readily achieved using purely organic compounds. This review examines metal-based therapeutics through a unifying conceptual framework that distinguishes between coordination complexes functioning primarily as inert structural scaffolds and those in which the metal centre serves as the dominant functional element. Scaffold-dominated complexes exploit kinetically stable coordination geometries to present ligands in rigid, stereochemically defined arrangements, enabling selective recognition of proteins and nucleic acids through shape complementarity and spatial control. In contrast, functional-centre-dominated complexes derive biological activity from metal-centred processes such as ligand exchange, redox cycling, catalysis, and photophysical activation, allowing dynamic and stimulus-responsive interactions with biological systems. The review highlights representative examples across both paradigms and emphasizes strategies that intentionally bridge structural stability with controlled activation at the target site. Emerging stimulus-responsive and multimodal approaches illustrate how metal complexes can be integrated into optimized therapeutic systems that align chemical reactivity with biological context and treatment modality. Collectively, this perspective underscores the unique structural and mechanistic space occupied by metal complexes in medicine and outlines design principles to guide the development of next-generation metallodrugs with improved selectivity, efficacy, and translational potential.

## Abstract 2

This review categorises metal-based therapeutics by their primary role, distinguishing between complexes acting as inert structural scaffolds for biomolecule recognition and those where the metal centre drives dynamic functional reactivity.

## Graphical abstract

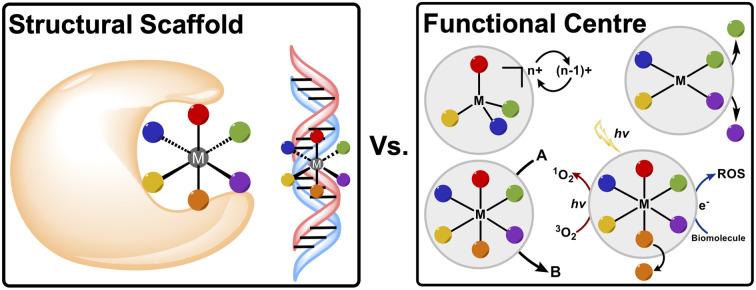

## Introduction

The therapeutic use of metal ions and metal complexes can be traced to ancient civilizations, where metal-containing remedies were routinely incorporated into medical practice. Compounds with gold, arsenic, mercury, copper, and iron were applied empirically to treat infections, inflammatory conditions, and chronic diseases, often without a clear understanding of their chemical composition or biological mechanisms.^1–4^ While these early remedies frequently suffered from severe toxicity and limited reproducibility, they nonetheless demonstrated that metals could exert profound biological effects, laying the groundwork for later, more systematic exploration of metal-based therapeutics.

The transition from empirically used metal-based remedies to rationally designed metallodrugs emerged in the early 20th century through the pioneering work of Paul Ehrlich. His development of the arsenic-containing compound arsphenamine, later termed Salvarsan, constituted the first major success of a structurally defined metal-based therapeutic agent.^5,6^ At a time when treatments for infectious diseases were scarce, Salvarsan exhibited selective toxicity toward *Treponema pallidum*, enabling effective treatment of syphilis. Crucially, this selectivity demonstrated that a drug could be designed to target a pathogenic organism while minimizing damage to the host. In this way, Salvarsan provided the first experimental confirmation of Ehrlich's magic bullet concept, the idea that therapeutic efficacy arises from precise chemical structures capable of selectively recognizing and attacking disease-causing pathogens. Despite its clinical success, the synthesis, formulation, and biological mode of action of Salvarsan were poorly understood for decades, and recent studies have shown that earlier hypotheses were incorrect.^5,6^ Advances in analytical and structural chemistry eventually revealed that Salvarsan is not a single molecular entity but a mixture of cyclic oligomeric arsenic species formed by 3-amino-4-hydroxyphenyl with As–As bonds. Mechanistic studies further suggested that the biologically active form is generated inside the living organism through slow oxidative transformation of the dominant oligomers.^7^

A decisive turning point in the field occurred several decades later with the discovery of *cis*-diamminedichloroplatinum(ii), commonly known as cisplatin.^8^ In 1965, Barnett Rosenberg observed that platinum electrodes used in experiments investigating the effects of electric fields on *Escherichia coli* produced electrolysis products that inhibited bacterial cell division.^9^ This serendipitous observation led to the isolation and identification of cisplatin and its subsequent evaluation in cancer models.^10^ Unlike earlier metal-based therapies, cisplatin demonstrated extraordinary efficacy in solid tumours, culminating in its FDA approval in 1978. Its clinical impact was particularly important in the treatment of testicular and ovarian cancers, transforming previously lethal diagnoses into highly curable diseases.^11–13^ The later elucidation of cisplatin's mechanism of action provided a foundational framework for understanding how metal coordination chemistry translates into therapeutic effect. Following cellular uptake, cisplatin undergoes aquation, generating reactive platinum species that form covalent adducts with DNA, predominantly through intrastrand crosslinks at guanine residues.^14–20^ These lesions distort the DNA helix, interfere with replication and transcription, and ultimately trigger apoptosis. This mechanistic clarity enabled the rational design of improved platinum-based drugs, including carboplatin, which features a more inert leaving group to reduce dose-limiting nephrotoxicity,^21^ and oxaliplatin, whose distinct ligand environment confers activity in tumours resistant to cisplatin and carboplatin.^22^ The success of cisplatin firmly established metal complexes as clinically viable and mechanistically sophisticated therapeutics, catalysing renewed interest in metals as active agents rather than merely toxic liabilities.^23–27^

**Fig. 1:**
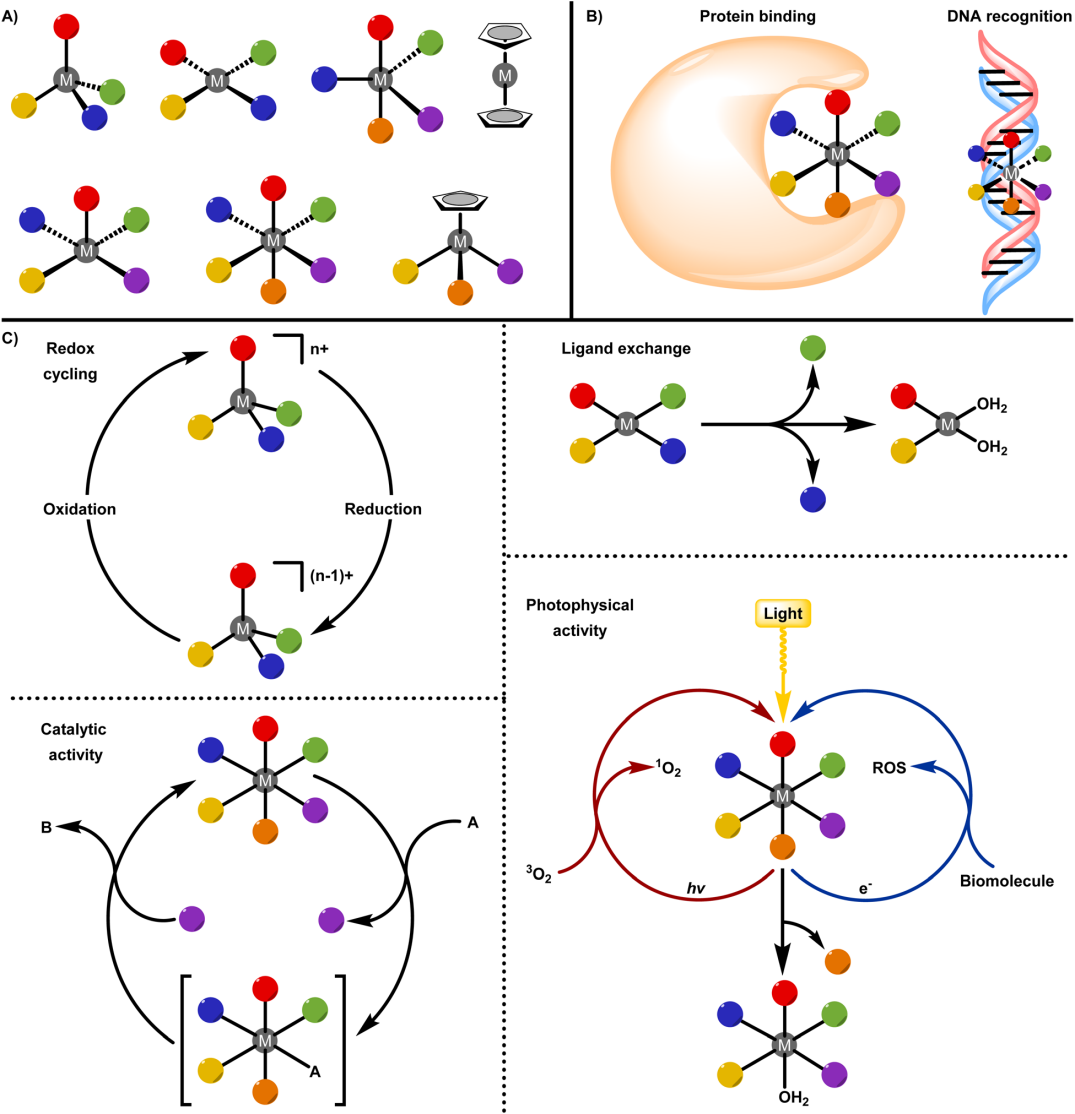
Representation of metal complexes as structural scaffolds and functional centres. (A) Square planar, trigonal bipyramidal, square pyramidal, octahedral, sandwich, and half-sandwich geometries of metal complexes. (B) The metal as a structural scaffold: protein binding and DNA recognition. (C) The metal as a functional centre: redox cycling, ligand exchange, catalytic activity, photophysical activity.

Importantly, cisplatin also reinforced a metallo-centric view of drug action, in which the metal ion plays a central role in determining reactivity, selectivity, and biological outcome.^28–41^ Building on this foundation, contemporary medicinal inorganic chemistry has expanded beyond platinum to encompass a diverse range of metals, including but not limited to ruthenium, rhenium, copper, iridium, and others.^42–55^ These systems exploit unique features of metal coordination chemistry, such as access to multiple oxidation states,^56–61^ tuneable ligand-exchange kinetics,^62–78^ catalytic activity,^79–87^ and photophysical properties,^62–74^ to achieve mechanisms of action inaccessible to purely organic drugs. In this review, metal complexes are classified according to whether the metal centre primarily serves as a chemically inert structural scaffold or functions as a reactive and biologically active centre (Fig. 1). This distinction provides a unifying framework for understanding how metal-based drugs are designed, how they interact with biological systems, and how their chemical properties influence therapeutic performance from molecular concept to clinical application.

## The metal as a structural scaffold

Within the inert structural scaffold paradigm, the metal centre is chosen primarily for its kinetic stability and ability to impose well-defined coordination geometries. In this capacity, the metal ion serves as an architectural element that enables coordination environments and stereochemical complexity that are fundamentally inaccessible to purely organic frameworks. Whereas organic scaffolds are largely confined to linear, trigonal planar, or tetrahedral geometries, transition-metal centres can support higher coordination numbers and diverse geometrical arrangements (Fig. 1A). This expanded structural repertoire allows substituents to be precisely positioned along multiple spatial axes, generating distinctive three-dimensional shapes that can enhance molecular recognition and promote selective interactions with biological targets (Fig. 1B).^88–97^

### Protein binding

**Fig. 2:**
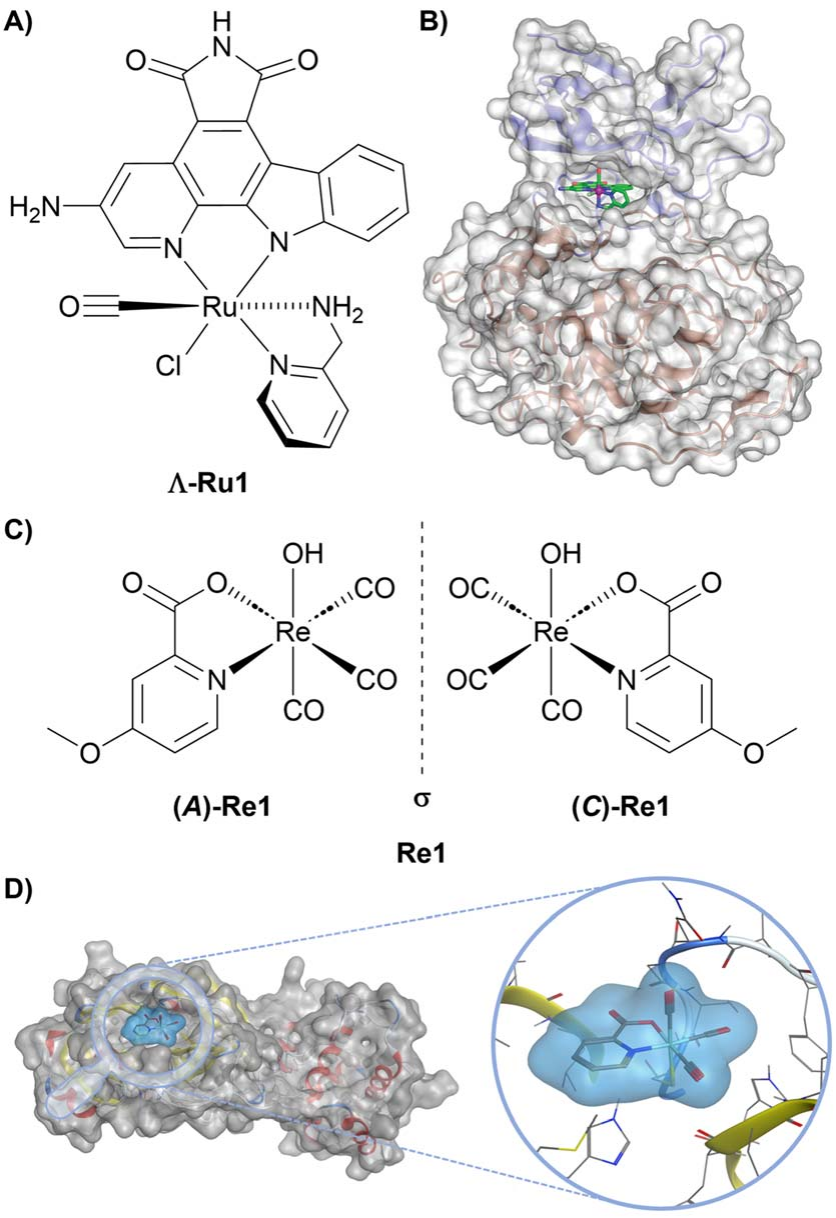
Representative metal complexes as protein inhibitors. (A) Chemical structure of Λ-Ru1. (B) Crystal structure of GSK3β with Λ-Ru1 bound to the ATP-binding (PDB: 1Q3D). Reprinted with permission from ref. 98 (L. Feng *et al.*, 2011), Copyright 2011 American Chemical Society. (C) Chemical structure of the racemic mixture of Re1, showing the (A)-Re1 and (C)-Re1 enantiomers. (D) Computationally predicted binding pose of the Re1 fragment bound to the thiol group of Cys145 of 3CL^pro^ (PDB: 6Y2F). Reproduced from ref. 99 (J. Karges *et al.*, 2023), licensed under CC BY-NC 3.0.

Octahedral metal complexes can function as rigid, three-dimensional scaffolds capable of mimicking the globular binding motifs commonly found in protein–ligand interactions. Such structurally enforced three-dimensionality is particularly advantageous for targeting proteins with well-defined yet subtly variable active sites, exemplified by kinases, acetylcholinesterase, trypsin, and thrombin, as well as for discriminating between structurally highly similar protein structures, where small differences in shape and chemical environment can be exploited to achieve high selectivity.^88–97^ A compelling demonstration of this strategy is provided by a series of octahedral Ru(ii) and Ir(iii) pyridocarbazole complexes developed as highly selective protein kinase inhibitors. Inspired by the natural product staurosporine, these compounds employ an inert metal centre as a structural template. In this design, the pyridocarbazole ligand engages the ATP adenine-binding region, while additional metal-coordinated ligands project into adjacent regions of the ATP-binding pocket. This spatial arrangement allows the complexes to simultaneously contact multiple structural elements of the kinase active site, thereby enhancing both affinity and selectivity. Within this series, the complex Λ-Ru1 (Fig. 2A) functions as a classical ATP-competitive inhibitor and exhibits exceptionally potent inhibition of glycogen synthase kinase 3α (GSK3α), with an inhibitory effect of 0.9 nM and selectivity exceeding 10^5^-fold over other kinases. The rigid octahedral architecture positions the complex within a conformationally sensitive region of the ATP-binding pocket (Fig. 2B), enabling interactions with the glycine-rich loop that are inaccessible to planar organic inhibitors such as staurosporine. The resulting globular shape and precisely oriented ligands promote extensive hydrophobic contacts across both the N-terminal and C-terminal domains and stabilize an open conformation of the glycine-rich loop.^98^ This example highlights how an inert metal centre can function as a purely structural element, enabling fine-tuned three-dimensional organization that translates directly into exceptional selectivity and potency in biological recognition.

The 3-chymotrypsin-like protease (3CL^pro^) is a critical enzyme in the SARS-CoV-2 replication cycle and a validated target for antiviral intervention. In this context, a series of Re(i) tricarbonyl complexes, particularly with a picolinic acid ligand, were reported as inhibitors of 3CL^pro^, exhibiting preliminary selectivity over human proteases and other SARS-CoV-2-associated enzymes. Among these, the racemic compound Re1 (Fig. 2C) demonstrated the most potent inhibition of 3CL^pro^, with an IC_50_ of 3.3 µM. Importantly, enantioselective separation of Re1 revealed a striking dependence of the biochemical activity on the chirality of the metal centre. The (A)-Re1 enantiomer exhibited substantially enhanced inhibitory potency (IC_50_ = 1.8 µM) compared to its enantiomer (C)-Re1, which displayed only weak inhibition (IC_50_ = 57 µM). Interestingly, these chirality-based discrepancy in activity was not only observed in the biochemical assessment but also during antiviral cellular studies. Molecular docking studies suggested that this difference arises from the three-dimensional ligand arrangement imposed by the metal centre. (A)-Re1 adopts a conformation that fits into the 3CL^pro^ active site, allowing the methoxy group on the picolinic acid ligand to occupy a complementary subpocket (Fig. 2D). By contrast, the geometry of (C)-Re1 produces steric clashes, preventing effective binding. These results highlight the pivotal role of absolute stereochemistry in determining the biological activity of metal complexes and emphasize that, when metal centres are used as rigid structural scaffolds, the spatial arrangement of ligands is a critical determinant of efficacy.^99^

### DNA recognition

Metal complexes can interact with DNA strands through a remarkably diverse array of non-covalent and coordinate binding modes that often exceed the capabilities of classical organic small molecules. They can interact with the negatively charged phosphate backbone through electrostatic attraction, insert themselves into the spaces between base pairs by intercalation, or fit into the major or minor grooves of the DNA helix through groove binding. Often, a single metal complex does not use just one mode; instead, it exhibits mixed or intermediate binding behaviour, where the type of interaction depends on properties of the metal, including its coordination number, geometry, overall charge, and the chemical nature of its ligands. These factors together determine how the metal complex fits and interacts with DNA.^100–103^

**Fig. 3:**
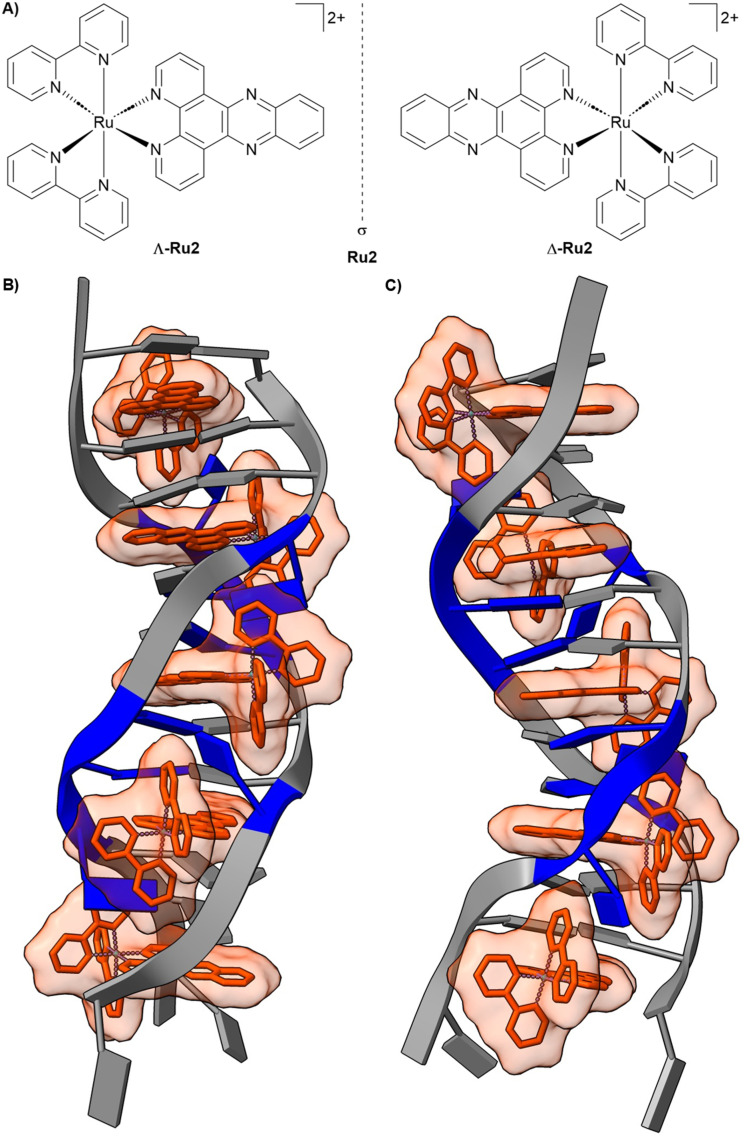
Representative metal complex as a DNA binder. (A) Chemical structure of the racemic mixture of [Ru(bpy)_2_(dppz)]^2+^ (Ru2), showing the Δ-Ru2 and Λ-Ru2 enantiomers. (B) Front view of Δ-[Ru(bpy)_2_(dppz)]^2+^ (Δ-Ru2) bond to the oligonucleotide 5′-C_1_G_2_G_3_A_4_A_5_A_6_T_7_T_8_A_9_C_10_C_11_G_12_-3′ (PDB: 4E1U). (C) 90° rotated view around the helix axis. Data originally reported by ref. 107 (H. Song *et al.*, 2012).

Ru(ii) polypyridyl complexes present a promising class of inert metal complexes that can act as a rigid structural scaffold for DNA recognition. The extensively studied [Ru(2,2′-bipyridine)_2_(dipyrido[3,2-*a*:2′,3′-*c*]phenazine)]^2+^ compound Ru2 (Fig. 3A) was initially characterized as a non-covalent DNA intercalator whose luminescence is “light-up” activated upon binding to duplex DNA. Early models described this behaviour as passive intercalation into well-matched B-form DNA, in which the insertion of the planar dppz ligand shields the complex from solvent quenching.^104,105^ More recent studies, however, have revealed a more nuanced and adaptive mode of DNA recognition. At destabilized sites, such as single-base mismatches or abasic lesions, Ru2 exhibits markedly enhanced luminescence and prolonged excited-state lifetimes relative to intact duplex regions. These photophysical responses correlate with the local thermodynamic instability of the DNA and are consistent with a metalloinsertion binding mode. The dipyrido[3,2-*a*:2′,3′-*c*]phenazine ligand inserts from the minor groove, partially replacing the destabilized base pair within the π-stacking framework. Importantly, this binding is dependent on the chirality of the metal complex. The Δ-enantiomer (Δ-Ru2) generally shows higher emission across B-form duplex DNA, reflecting tighter binding to the right-handed helix, and displays particularly strong luminescence at single-base mismatches (Fig. 3B), accompanied by long-lived excited-state components indicative of enhanced solvent shielding in a minor-groove insertion geometry. Conversely, Λ-Ru2 exhibits enhanced emission primarily at abasic sites, highlighting that absolute configuration can bias defect preference and tune the photophysical output. This defect-selective recognition is strikingly distinct from the behaviour of conventional organic intercalators and groove binders.^106,107^ These observations demonstrate that metal complexes can discriminate between subtle structural features of DNA. The metal centre functions as a structural scaffold, enforcing precise ligand orientation, while simultaneously enabling a sensitive functional response through its photophysical properties. This dual capability, shape-based recognition combined with tuneable signalling, underscores the unique potential of metal complexes to probe and manipulate nucleic acid structure in ways that are inaccessible to traditional organic molecules.

## The metal as a structural scaffold

In the functional-centre paradigm, the metal ion itself serves as the primary active element, with its intrinsic electronic, redox, and physicochemical properties dictating the biological outcome. Within this framework, metallodrugs exert their effects through mechanisms that are directly centred on the metal (Fig. 1C). Key metal-centred processes that drive these actions include redox cycling,^56–61^ ligand exchange,^62–78^ catalytic turnover,^79–87^ and photophysical reactivity.^62–74^ Crucially, these mechanisms can be selectively harnessed by exploiting the unique chemical environment of diseased or stressed cells. For example, pathological microenvironments often feature altered redox homeostasis, localized pH gradients, or differential substrate availability, which can trigger or enhance metal-centred reactivity, thereby conferring specificity under intracellular conditions.^58^ By integrating the intrinsic reactivity of the metal with the spatial and chemical context of the target site, this paradigm enables metallodrugs to achieve potent and context-dependent biological effects, distinguishing them from conventional organic therapeutics that rely primarily on passive binding interactions.

### Redox cycling

**Fig. 4:**
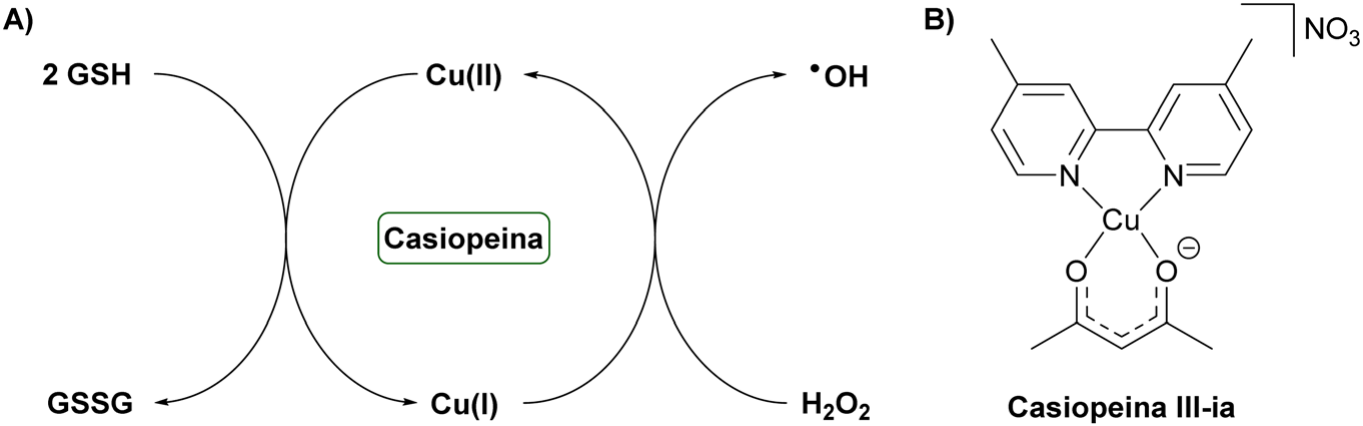
Representative example of a redox cycling metal complex. (A) Proposed mechanism of action of the Casiopeina family by redox cycling. (B) Chemical structure of Casiopeina III-ia.

Systemic anticancer therapy continues to be limited by insufficient selectivity and the frequent development of drug resistance. Many conventional chemotherapeutics act primarily by exploiting differences in proliferation rates between cancerous and healthy cells rather than targeting tumour-specific molecular vulnerabilities. This approach often leads to dose-limiting toxicities in normal tissues and reduced long-term efficacy due to intrinsic or acquired resistance mechanisms, such as target mutations, enhanced drug efflux, pathway reprogramming, and altered cellular metabolism.^108,109^ Within this therapeutic landscape, cellular redox homeostasis has emerged as a mechanistically distinct and promising target. Although redox balance is tightly regulated under normal conditions, malignant cells frequently maintain elevated basal levels of reactive oxygen species (ROS) due to altered metabolism and mitochondrial dysfunction. This elevated oxidative stress renders cancer cells more vulnerable to further perturbations, meaning that strategies designed to induce ROS accumulation or deplete antioxidant defences can preferentially affect tumour cells over healthy cells.^110,111^ Redox-active metal complexes are highly promising candidates for this approach, as the metal centre itself can function as a catalytic cycling element that directly modulates intracellular redox processes.^56–61^ A prototypical example of this strategy is provided by the Casiopeina family of Cu(ii) complexes, which exploit redox chemistry to elicit anticancer effects. Casiopeinas are mixed-chelate Cu(ii) complexes that combine an aromatic diimine ligand, commonly 1,10-phenanthroline or 2,2′-bipyridine, with a secondary O,O- or N,O-chelating ligand, such as acetylacetonate, aminoacidato, or salen-type donors. These coordination environments are deliberately labile and electronically tuneable, allowing controlled redox cycling of the copper center.^112,113^ Casiopeinas operate *via* Cu(ii)/Cu(i) redox cycling, in which Cu(ii) is reduced to Cu(i) by glutathione, generating glutathione radicals that dimerize to form oxidized glutathione disulfide (Fig. 4A). Simultaneously, Cu(i) can react with hydrogen peroxide to regenerate Cu(ii), producing highly reactive hydroxyl radicals that induce cellular damage.^114–117^ Among this family, Casiopeina III-ia (Fig. 4B) has advanced to Phase I clinical trials in Mexico due to its favourable balance of potency and tolerability. Structurally, the Casiopeina III-ia complex incorporates a 4,4′-dimethyl-2,2′-bipyridine ligand and an acetylacetonate ligand, adopting a distorted square-planar geometry.^118^*In vitro*, it exhibits pronounced cytotoxicity across multiple cancer cell lines, including rat glioma cells (EC_50_ ≈ 15 µg mL^−1^)^119^ and HCT-15 colon carcinoma cells (EC_50_ ≈ 10 µg mL^−1^),^120^ while sparing normal lymphocytes.^121^ Mechanistically, Casiopeina III-ia elevates intracellular ROS, inducing oxidative DNA damage and mitochondrial dysfunction that disrupts membrane potential and ultimately triggers cell death. At the signalling level, the complex activates a ROS-dependent c-Jun N-terminal kinase (JNK) pathway, linking redox stress to apoptotic and autophagic outcomes.^119^*In vivo*, Casiopeina III-ia demonstrates antitumor efficacy in nude mice bearing HCT-15 xenografts, suppressing tumour proliferation and promoting apoptosis.^120^ Collectively, these findings establish Casiopeina III-ia as a multitarget, redox-active metallodrug whose therapeutic effects arise from a combination of DNA breakage, mitochondrial disruption, and ROS-dependent JNK signalling, with apoptosis serving as the principal mode of cell death.

### Ligand exchange

**Fig. 5:**
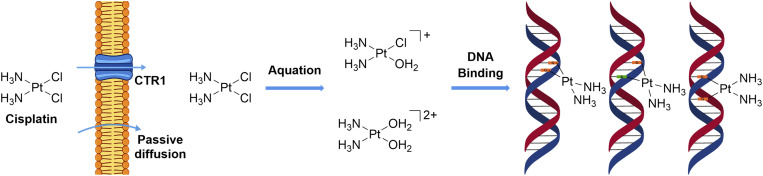
Chemical structure of cisplatin and its mechanism of cellular uptake, aquation, and DNA binding.

Ligand exchange kinetics are a central consideration for metal complexes with a functional metal centre because they determine the balance between stability during transport and activation at the biological target. A coordination complex must remain intact long enough to reach its site of action, yet be sufficiently labile to engage with biomolecules. The ligand exchange behaviour of a metal complex is highly sensitive to its coordination environment. Small changes in coordination geometry, ligand donor type, or steric hindrance can drastically alter both the rate and mechanism of substitution, leading to significant differences in biological activity. Excessive lability can cause premature ligand dissociation and off-target reactions with abundant extracellular nucleophiles, while excessive inertness can hinder activation and reduce effective target engagement. Designing effective metallodrugs, therefore, requires careful kinetic tuning, aligning substitution rates with biologically relevant timescales to optimize therapeutic efficacy. Numerous examples of metal complexes exploiting ligand exchange for medicinal purposes have been described in the literature.^43,53,75–78,122–124^ A prototypical and clinically significant example is cisplatin (Fig. 5), whose pharmacological activity is intimately linked to its ligand exchange properties. In the bloodstream, the high chloride concentration (∼100 mM) stabilizes the neutral dichloride complex, suppressing premature hydrolysis and maintaining the complex in its transport form. Cisplatin enters cells primarily by passive diffusion and to a lesser extent through the copper transporter CTR1. Once inside the cell, the lower intracellular chloride concentration promotes aquation, converting cisplatin into more electrophilic species capable of covalent interactions with DNA. The first aquation event produces the monoaqua monochloro complex [Pt(NH_3_)_2_Cl(OH_2_)]^+^, and a subsequent aquation can generate the diaqua species [Pt(NH_3_)_2_(OH_2_)_2_]^2+^. Both of these activated forms are highly reactive toward nucleophilic sites on DNA. Cisplatin primarily targets the N7 position of guanine and, to a lesser extent, adenine, forming covalent adducts that include both intrastrand and interstrand crosslinks. Intrastrand lesions arise from platinum bridging adjacent guanines on the same strand, whereas interstrand cross-links form when guanines on opposite strands are linked (Fig. 5). These adducts induce local DNA distortions, including bending and unwinding of the duplex, which block the progression of DNA and RNA polymerases, thereby interfering with replication and transcription. These disruptions activate DNA damage response pathways, leading to cell-cycle arrest and, ultimately, apoptosis.^19,125–129^ This example highlights that precise control of ligand exchange kinetics is a critical determinant of metallodrug pharmacology, governing not only stability and transport but also the timing and specificity of target engagement.

### Catalytic activity

**Fig. 6:**
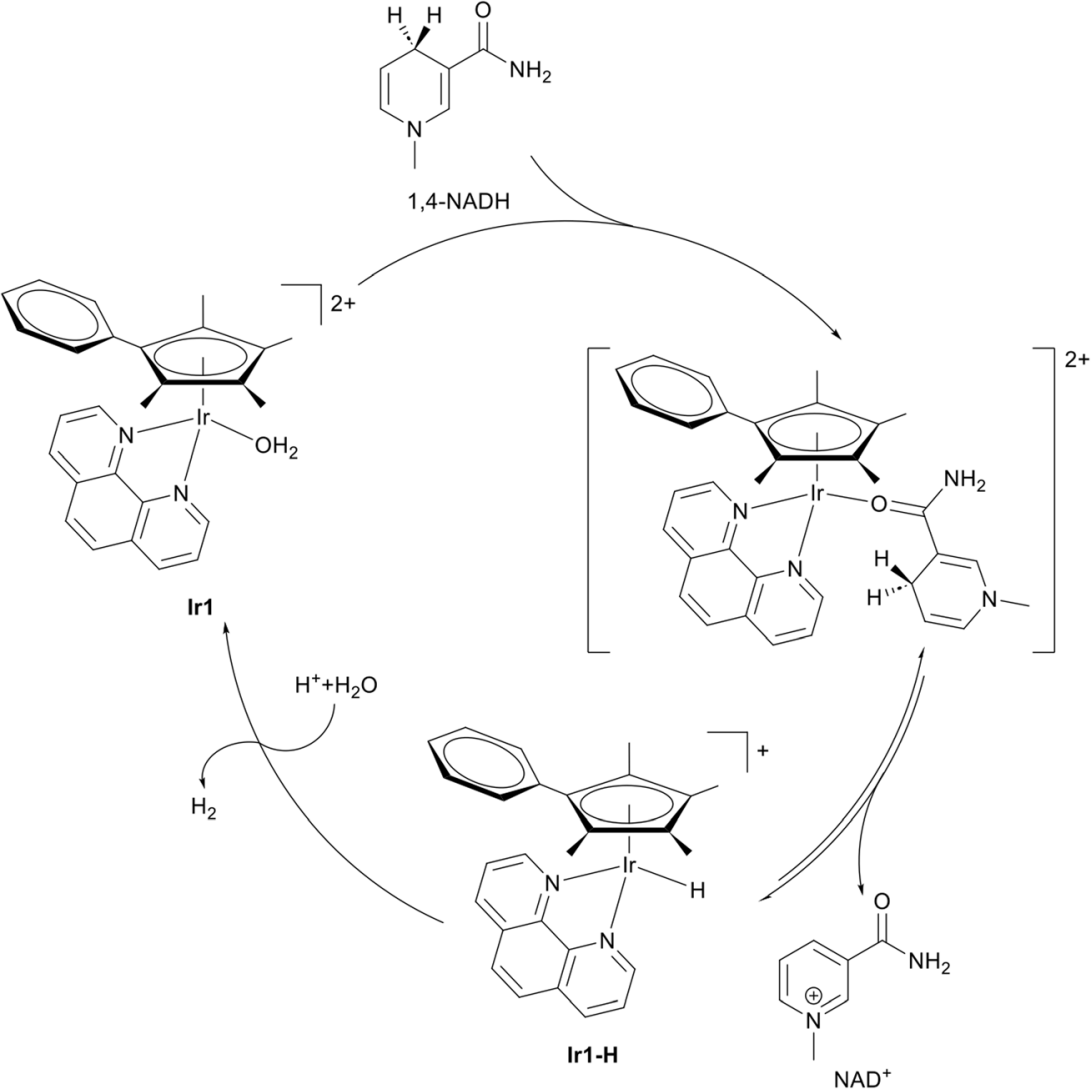
Chemical structure of [(η^5^-C_5_Me_4_C_6_H_5_)Ir(phen)(H_2_O)]^2+^ (Ir1) and its catalytic conversion of NADH to NAD^+^ by hydride transfer.

The catalytic activity of metal complexes represents a distinct and increasingly important paradigm in medicinal inorganic chemistry. Catalytic metallodrug strategies are typically classified into several major functional classes, including enzyme mimics, catalytic generation of ROS, catalytic degradation of biomolecules, and non-physiological catalytic reactions within cells, such as C–C cross-coupling, cycloadditions, hydrogenation, transfer hydrogenation, thiol oxidation, and functional-group deprotection.^79–87^ By harnessing catalytic cycles, these approaches can amplify biological effects, enabling potent therapeutic activity at comparatively low drug concentrations. Exemplary, organometallic complexes have been previously described to catalyse the conversion of NADH to NAD^+^ by hydride transfer within biological environments.^130–132^ A representative compound [(η^5^-C_5_Me_4_(C_6_H_5_))Ir(1,10-phenanthroline)(H_2_O)]^2+^Ir1 (Fig. 6), illustrates this concept. Ir1 features a catalytically competent Ir(iii) centre supported by a Cp*-type arene-substituted cyclopentadienyl ligand and a chelating phenanthroline ligand. In an aqueous solution, Ir1 catalyses the oxidation of 1,4-NADH to NAD^+^ through an Ir(iii) hydride intermediate (Ir1–H) within 10 minutes. Over 33 hours, the Ir1–H signal gradually diminishes while the solution pH rises, consistent with protonation of the metal hydride, H_2_ evolution, and regeneration of the active Ir1 catalyst (Fig. 6). Under physiological conditions, Ir1 achieves a turnover number of 75 after 24 hours and a turnover frequency of up to 4.3 h^−1^ for NADH oxidation. In A2780 human ovarian cancer cells, Ir1 elevated the intracellular NAD^+^/NADH ratio from 8.0 to 14.9 within 6 hours, consistent with a potent cellular oxidative effect.^130^ These observations suggest that Ir1 can act as a redox-modulating agent, potentially perturbing ROS balance and inducing oxidative stress to achieve therapeutic effects in cancer cells.^131^ Collectively, these findings highlight organometallic Ir(iii) cyclopentadienyl complexes as promising examples of catalytic metallodrugs, capable of amplifying redox-mediated anticancer mechanisms through sustained intracellular catalysis. By linking catalytic turnover directly to biological outcomes, such complexes exemplify a powerful strategy for designing next-generation metallopharmaceuticals that combine chemical precision with therapeutic amplification.

### Photophysical activity

**Fig. 7:**
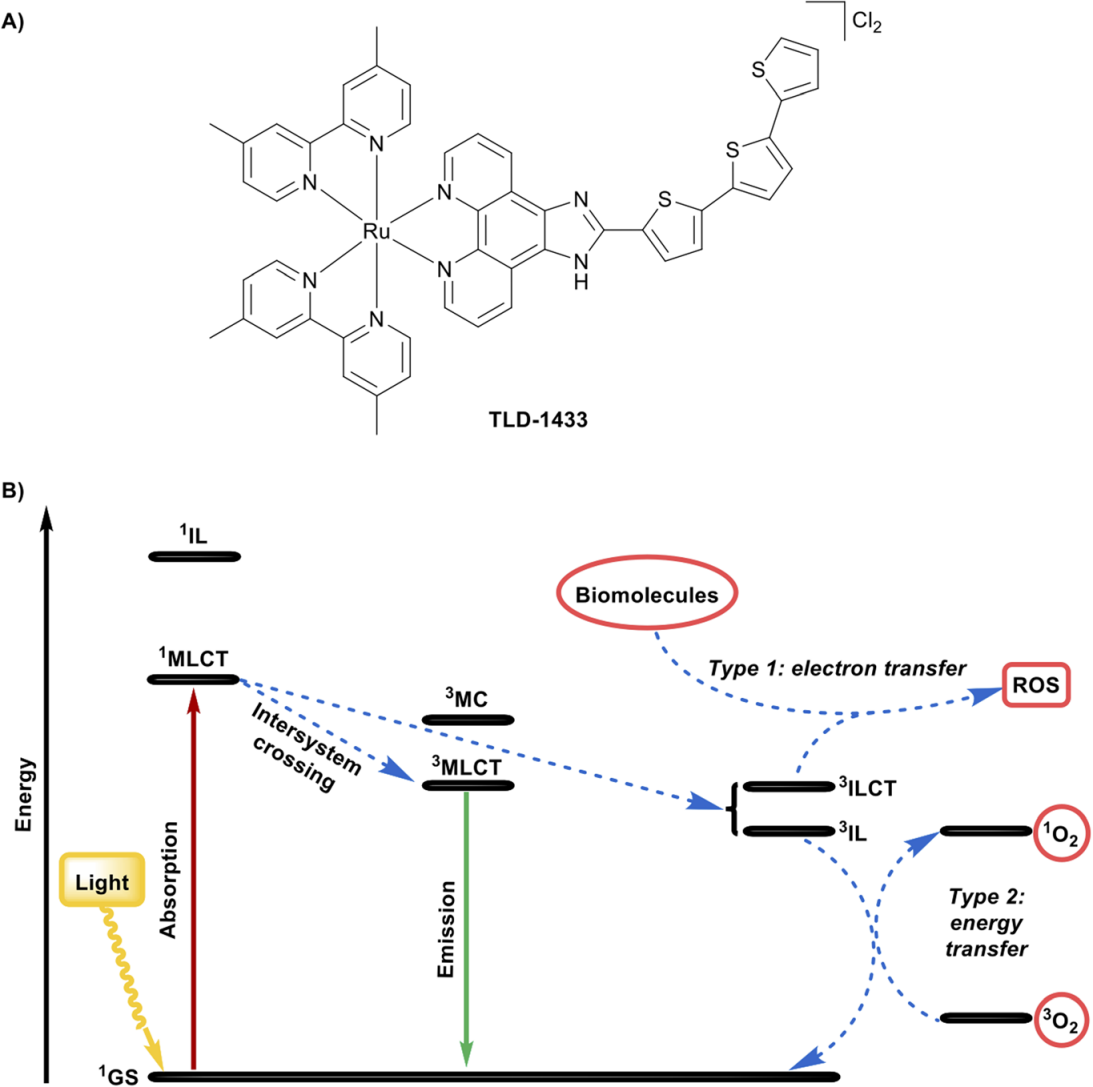
Representative example of a metal complex with photophysical properties in PDT. (A) Chemical structure of TLD-1433. (B) Mechanism of action of TLD-1433 upon visible light irradiation.

Certain transition-metal complexes are particularly effective as photosensitizers because the heavy-atom effect of the metal centre enhances spin–orbit coupling, promoting efficient intersystem crossing from singlet to long-lived triplet states. This enables a greater population of reactive triplet excited states than is typically possible with purely organic photosensitizers, increasing the efficiency of photochemical processes such as singlet oxygen generation and electron-transfer reactions. These properties enable light to act as an external trigger, controlling when and where biological activity occurs. Recent advances in this field have focused on metal-based agents for photodynamic therapy and photoactivated chemotherapy, highlighting both opportunities and challenges for clinical translation.^62–74^ The effectiveness of such agents depends not only on absorption within the therapeutic window (600–800 nm) but also on precise tuning of the nature and lifetime of the excited triplet state. These parameters can be modulated by metal choice, oxidation state, and ligand design, which allow access to metal-to-ligand charge-transfer, ligand-to-metal charge-transfer, intra-ligand, or metal-centred states.^62,67,68^ Photodynamic therapy requires a photosensitizer, molecular oxygen, and light of an appropriate wavelength. Upon absorption of light, the photosensitizer is promoted from its ground singlet state to an excited singlet state, which can undergo intersystem crossing to form a long-lived triplet state. From this triplet state, the photosensitizer can react through two main pathways. In a type I reaction, the photosensitizer transfers an electron to nearby biomolecules, generating radical species, whereas in type II reactions, energy is transferred to ground-state triplet oxygen to produce highly reactive singlet oxygen. After reaction, the photosensitizer returns to the ground state, allowing multiple excitation cycles and catalytic-like efficacy.^65,68^ A leading example of this approach is TLD-1433 (Fig. 7A), the first ruthenium-based photosensitizer to enter clinical trials. TLD-1433 emerged from a series of Ru(ii) α-oligothienyl dyads optimized for aqueous solubility, triplet-state lifetime, and light-triggered potency.^133^ The racemic monometallic Ru(ii) dyad features an ionizable imidazo[4,5-*f*][1,10]phenanthroline ligand carrying an α-terthienyl organic chromophore, two 4,4′-dimethyl-2,2′-bipyridine co-ligands. The design of TLD-1433 favours a long-lived intra-ligand state by targeted π-extension along the Ru–N axis, extending triplet lifetime while avoiding dissociative metal-centred states. Its bis-heteroleptic architecture, one π-extended ligand and two 4,4′-dimethyl-2,2′-bipyridine co-ligands, enhances solubility, limits aggregation, and supports formulation stability. The extended phenanthroline ligand allows modular chromophore installation and pH-dependent charge tuning, promoting selective interactions with cancer cell membranes. The α-terthienyl chromophore accesses low-energy terthienyl-centred intra-ligand states, with the Ru(ii) metal-to-ligand charge-transfer state acting as an antenna to capture longer-wavelength visible light (Fig. 7B). Following excitation, the excited singlet metal-to-ligand charge-transfer state rapidly relaxes to the lowest-energy excited triplet metal-to-ligand charge-transfer state, aligning triplet-state energetics with the tissue optical window and enabling efficient singlet oxygen production. The α-terthienyl ligand also supports the formation of an intramolecular excited triplet intra-ligand charge-transfer state, which can engage in electron transfer with biomolecules, potentially amplifying photochemical reactivity.^65,68^*In vitro*, TLD-1433 exhibits potent, light-dependent cytotoxicity across multiple cancer cell lines (HL-60: EC_50,light_ = 7.2 µM; SK-MEL-28: EC_50,light_ = 2.3 µM) while remaining non-toxic in the dark (EC_50,dark_ > 100 µM), confirming that its activity is directly linked to triplet-state ROS generation.^65,133^ In the Phase I intravesical trial for Bacillus Calmette-Guérin-unresponsive nonmuscle-invasive bladder cancer, TLD-1433 was instilled into the bladder for approximately 60 minutes, rinsed, and then illuminated using a central spherical diffuser delivering 520 nm light (90 J cm^−2^) over 1–1.5 hours. The treatment was safe and well-tolerated, with no systemic phototoxicity, and showed early efficacy, with 2 of 3 patients achieving a durable complete response lasting 18 months after a single treatment. Pharmacokinetic analysis demonstrated rapid clearance from both urine and plasma, supporting repeat treatments and minimal systemic exposure.^65,68,134^ TLD-1433 is currently being evaluated in a Phase II clinical trial for intravesical photodynamic therapy in Bacillus Calmette-Guérin-unresponsive or intolerant nonmuscle-invasive bladder cancer patients (clinicaltrials.gov (http://clinicaltrials.gov) identifier: NCT03945162).^66,68^ Collectively, TLD-1433 exemplifies how metal-based phototherapeutic design integrates photophysical control, ROS generation, and cellular targeting to achieve precise, light-triggered anticancer effects, illustrating the broader potential of transition-metal complexes in clinical photomedicine.

**Fig. 8:**
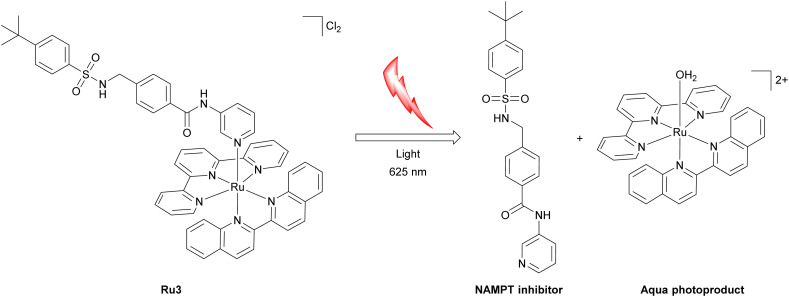
Chemical structure of Ru3 and its photoactivated mechanism of action.

As a complementary approach to photodynamic therapy, increasing research effort has been devoted to photoactivated chemotherapy that combines photoactivation with ligand release kinetics. In photoactivated chemotherapy, irradiation converts an otherwise inert metal complex into a cytotoxic species through light-driven chemical transformations such as photosubstitution, photoinduced electron transfer, release of bioactive ligands, or ligand-centred photocleavage. These processes operate predominantly in the absence of molecular oxygen, enabling them to function in the hypoxic microenvironment of tumors.^62,70,135^ Prominent examples of photoactivated chemotherapy are Ru(ii) polypyridyl complexes such as [Ru(2,2′:6′,2′′-terpyridine)(2,2′-biquinoline)(inhibitor structure)]^2+^ (Fig. 8) bearing a pyridine-functionalized nicotinamide phosphoribosyltransferase inhibitor as a monodentate ligand. The complex combines high dark stability with efficient photosubstitution under clinically relevant red light. *In vitro*, Ru3 exhibits low dark toxicity toward multiple cancer cell lines under normoxic conditions, but becomes markedly cytotoxic upon irradiation. Notably, the observed phototoxicity is preserved under hypoxic conditions, indicating that oxygen depletion does not compromise photocytotoxicity. Mechanistic studies show that photoactivation proceeds by population of triplet metal-to-ligand charge-transfer states with access to dissociative triplet metal-centred character, leading to light-induced release of the nicotinamide phosphoribosyltransferase inhibitor (Fig. 8). In contrast, singlet oxygen generation is negligible, confirming that phototoxicity arises primarily from ligand dissociation rather than reactive oxygen species formation. Consistent with this mechanism, Ru3 displays weak nicotinamide phosphoribosyltransferase inhibition in the dark (EC_50,dark_ = 4.8 µM), which is converted into potent enzyme inhibition upon irradiation (EC_50,light_ = 0.26 µM, 625 nm, 20.6 J cm^−2^) due to quantitative release of the active inhibitor, ultimately triggering NAD^+^ depletion and metabolic collapse. Crucially, because activation relies on oxygen-independent photosubstitution rather than singlet-oxygen generation, the phototoxicity of Ru3 is maintained under hypoxic conditions.^136^ Combined, these examples highlight how the concept of photoactivation and ligand release can function together.

## Emerging approaches

### Tumour-targeting metal complexes

While traditional metal-based drugs, such as cisplatin, are frequently hindered by poor pharmacokinetics and systemic toxicity, the development of targeted metal–complex conjugates has emerged as a sophisticated strategy to enhance selectivity. This precision is increasingly achieved by tethering metal centres to specific biological ligands that exploit receptor-mediated pathways to fulfil the unique biochemical requirements of tumour cells. For instance, sugar-based targeting, or glycoconjugation, leverages the “Warburg effect” to facilitate increased drug uptake by overexpressed transporters.^137–140^ In a similar vein, vitamin conjugation exploits the high demand of malignant cells for exogenous supplements, such as vitamins B6 and B7.^37,141–143^ For even greater therapeutic precision, antibody–drug conjugate bind to cancer-specific, overexpressed, or deregulated antigens, such as oestrogen and epidermal growth factor receptors. These systems often utilize the metal complex as a prodrug that remains inactive until it reaches its intended destination.^144–148^ Furthermore, peptide conjugation enables both precise cellular targeting and selective subcellular localization, significantly improving the ability of metal complexes to disrupt organelle-specific homeostasis.^149–153^ Beyond direct conjugation, the encapsulation of these complexes within nanocarriers, such as polymeric micelles or liposomes, shields them from premature degradation. These delivery systems utilize the enhanced permeability and retention effect, where “leaky” tumour vasculature and impaired lymphatic drainage allow nanoparticles to accumulate preferentially at the lesion site. Recent designs have further refined this approach by targeting the tumour microenvironment; by employing pH-sensitive or enzyme-triggered linkers, researchers ensure that the active metal species is released only under the acidic or enzyme-rich conditions found specifically within the tumour mass.^86,154–157^ A remarkable example of this strategy is encapsulation in human serum albumin, which delivers higher drug payloads to the tumour site without exacerbating systemic side effects.^158–161^ Collectively, these strategies represent a significant shift toward precision medicinal chemistry, effectively transforming traditional metallodrugs into “smart” therapeutic agents.

### Multimetallic complexes

Although combination therapy based on co-administration of separate drugs is common in oncology,^162–165^ it is often limited by mismatched pharmacokinetics. Independent agents typically differ in clearance, uptake routes, and bioavailability, leading to temporal and spatial desynchronization that prevents the intended drug ratio from reaching the same cell simultaneously.^166–168^ Covalent coupling overcomes this limitation by enforcing pharmacokinetic alignment, delivering both metal centres as a single entity with shared biodistribution and coordinated uptake. By engaging multiple cellular targets in parallel, such bimetallic architectures can increase the barrier to resistance by concurrently perturbing genetic and metabolic pathways.^73,169–180^ A compelling example is a heterobimetallic Ru(ii)–Pt(iv) conjugate designed to overcome platinum resistance by integrating cancer-activated chemotherapy with long-wavelength photodynamic therapy in a single molecule. In this construct, a thermally stable Pt(iv) prodrug is axially functionalized with both a histone deacetylase inhibitor (phenylbutyrate) and a Ru(ii) polypyridyl photosensitizer, linked by a flexible aliphatic spacer that preserves the independent functions of each metal. After energy-dependent endocytosis, intracellular reduction of the Pt(iv) centre in cancer cells releases a cisplatin-like Pt(ii) species that damages nuclear DNA, while phenylbutyrate promotes chromatin decondensation. In parallel, the Ru(ii) unit retains strong absorption in the biological window and efficiently generates singlet oxygen upon irradiation, providing an orthogonal, externally triggered cytotoxic pathway. Subcellular fractionation confirmed functional divergence after activation, with Pt accumulating in the nucleus and Ru localizing predominantly in the Golgi apparatus. The conjugate displays micromolar cytotoxicity in the dark and nanomolar potency upon visible-light irradiation, inducing apoptosis and paraptosis. Importantly, activity is retained in cisplatin- and doxorubicin-resistant ovarian cancer cells and in 3D tumour spheroids, underscoring how covalent integration of complementary metallodrugs can cooperate to overcome therapeutic resistance.^181^ This example illustrates how combining multiple metal complexes can overcome limitations inherent to a single molecular agent.

### Immune-activating metal complexes

Immune-activating metal complexes couple localized tumour cell killing with a systemic antitumor immune response by exemplary induction of immunogenic cell death. In this process, dying cancer cells expose or release damage-associated molecular patterns that recruit and instruct antigen-presenting cells. Key signals include calreticulin exposure as an “eat-me” cue,^182,183^ ATP secretion as a “find-me” signal,^184^ and release of high-mobility group box 1 protein, which activates innate immune pathways through pattern-recognition receptors.^185,186^ Together, these events promote dendritic cell maturation and subsequent T-cell priming.^187,188^ Two mechanistic classes of immunogenic cell death inducers are particularly relevant to metallodrug design. Type I inducers act primarily outside the endoplasmic reticulum, with endoplasmic reticulum stress arising secondarily from the primary cytotoxic lesion. In contrast, type II inducers localize to the endoplasmic reticulum and directly generate oxidative endoplasmic reticulum stress, engaging protein kinase RNA-like endoplasmic reticulum kinase-dependent signalling to initiate the immunogenic cell death cascade.^189^ Metal complexes are uniquely well-suited as immunogenic cell death platforms because their tuneable catalytic, redox, and photoactivation properties ensure high endoplasmic reticulum stress generation. To date, immunogenic cell death induction has been reported for many types of metal complexes.^177,189–191^ Among the most advanced examples is the Ru(iii) complex BOLD-100 (formerly known as IT-139, NKP-1339, and KP1339), which has progressed into clinical trials as a chemotherapeutic agent. BOLD-100 localizes to the endoplasmic reticulum and induces type II immunogenic cell death in colon adenocarcinoma cells, as demonstrated by calreticulin translocation, ATP release, and high-mobility group box 1 protein secretion.^192,193^ A combination of BOLD-100 and FOLFOX (folinic acid, fluorouracil, and oxaliplatin) is currently in Phase 1b/2a clinical trial for advanced gastrointestinal malignancies (clinicaltrials.gov identifier: NCT04421820).

### Ultrasound activating metal complexes

Ultrasound-activatable metal complexes extend stimulus-responsive anticancer therapy to deep tissue, circumventing the limited penetration of light-based activation. Current advances centre on ultrasound-triggered ligand release and sonodynamic therapy. In sonodynamic therapy, ultrasound activates a sonosensitizer to generate ROS through a combination of oxidative, mechanical, and thermal effects, with acoustic cavitation playing a central role.^194,195^ Cavitation involves the formation and collapse of microbubbles,^196^ producing intense mechanical forces that disrupt cellular membranes and organelles.^197,198^ Simultaneously, localized high-temperature “hot spots” generated during bubble collapse promote pyrolysis and sonoluminescence,^196,199^ populating excited states in suitable sonosensitizers. These excited states enable both type I electron–transfer pathways that generate radical species and type II energy–transfer pathways that yield singlet oxygen, with ROS generally regarded as the dominant cytotoxic output.^200,201^ Metal complexes are emerging as particularly effective sonosensitizers because heavy metal centres enhance spin–orbit coupling and intersystem crossing, facilitating access to long-lived triplet states that favour ROS generation. An increasing number of studies report on various types of metal complexes with the ability to interact *via* ultrasound-activated anticancer therapy.^202–213^ An example of this concept is a cyanine-based Re(i) tricarbonyl complex that combines sonodynamic therapy with gas therapy by generating singlet oxygen and releasing carbon monoxide upon ultrasound exposure. The complex displays minimal dark toxicity in breast cancer cells but potent ultrasound-activated cytotoxicity. Mechanistic studies revealed glutathione depletion, downregulation of glutathione peroxidase 4, lipid peroxidation, and cell death by ferroptosis, highlighting the potential application of metal complexes for ultrasound-induced anticancer therapy.^214^

## Conclusions and future perspectives

This review has organized metal-based medicinal agents into two overarching design paradigms: inert coordination complexes that function primarily as structural scaffolds, and complexes in which the metal centre itself serves as the dominant functional element. This distinction provides a coherent framework for rationalizing how coordination chemistry can be exploited to achieve biological activity that extends beyond the capabilities of conventional organic molecules.

In scaffold-dominated systems, kinetically inert metal centres enable the presentation of ligands in rigid, three-dimensional geometries that are difficult to access using purely organic frameworks. Protein inhibitors and DNA binders exemplify how well-defined coordination geometries, stereochemical control, and shape persistence can drive selective molecular recognition in complex biological environments. In these cases, the metal does not directly participate in chemical transformation, but instead acts as an architectural element that enforces spatial organization, enhances binding specificity, and enables recognition modes that are otherwise challenging to realize with flexible organic scaffolds.

In contrast, functional-centre-dominated complexes derive their biological activity from metal-centred reactivity. Ligand exchange processes, redox cycling, catalytic transformations, and photophysical responses allow these systems to interact dynamically with biological targets and microenvironments. Such behaviours enable controlled activation, signal amplification, and stimulus-responsive therapeutic action. Importantly, these reactivity profiles, particularly the combination of tuneable kinetics, multiple accessible oxidation states, and long-lived excited states, are inherently difficult to replicate with organic compounds, highlighting the distinctive mechanistic space occupied by metal-based agents.

While presented as conceptually distinct, these two paradigms frequently converge in effective therapeutic designs. Many successful metal complexes integrate inert structural features that ensure stability and selective transport with metal-centred activation mechanisms that are triggered only at the target site. Stimulus-responsive strategies, including light-, redox-, and ultrasound-activated systems, exemplify this convergence by coupling scaffold stability with precisely controlled functional activation. Clinical and preclinical advances in photodynamic and photoactivated chemotherapy further illustrate how metal complexes can be embedded within treatment paradigms that co-optimize molecular design, activation modality, and biological context.

Looking forward, continued progress in the field will depend on developing predictive structure–function relationships that link coordination geometry, stereochemistry, and electronic properties to biological performance *in vivo*. Future efforts should emphasize responsiveness to tumour microenvironmental cues, integration with combination therapies, and formulation strategies that preserve metal-centred function while optimizing pharmacokinetics. Equally important will be standardized reporting of chemical form, activation conditions, and biological context to enable meaningful comparison across studies. Taken together, these directions reinforce the view that metal complexes occupy a distinct and complementary therapeutic space, offering structural and functional capabilities that are not readily achievable with purely organic drugs.

## Author contributions

N. M. contributed to conceptualization, investigation, and writing—original draft. J. K. contributed to writing—review and editing by refining the manuscript structure and critically revising the content for clarity and language. All authors approved the final version of the manuscript.

## Conflicts of interest

There are no conflicts to declare.

## Data Availability

No primary research results, software or code have been included and no new data were generated or analysed as part of this review.
